# Antenatal management of *HNF4A*-MODY and *INSR* mutations in pregnancy

**DOI:** 10.1210/jcemcr/luag090

**Published:** 2026-04-29

**Authors:** Katherine Wu, Hamish Russell, Natassia Rodrigo

**Affiliations:** Department of Endocrinology, Nepean Hospital, Kingswood, New South Wales 2747, Australia; Diabetes and Endocrinology Department, Liverpool Hospital, Liverpool, New South Wales 2170, Australia; Conjoint Senior Lecturer, South West Clinical School, University of New South Wales, Liverpool, New South Wales 2170, Australia; Department of Endocrinology, Royal North Shore Hospital, St Leonards, New South Wales 2065, Australia; Senior Clinical Lecturer, Northern Clinical School, University of Sydney, St Leonards, New South Wales 2065, Australia

**Keywords:** maturity-onset diabetes of the young, type 1, hepatocyte nuclear factor 4, INSR protein, human, pregnancy in diabetics, prenatal care, fetal macrosomia, neonatal hypoglycemia

## Abstract

We report the case of a pregnant woman with coexisting pathogenic hepatocyte nuclear factor 4 alpha (*HNF4A)* and insulin receptor (*INSR)* gene variants. A 25-year-old lean Caucasian gravida 1, para 0 (G1P0) woman with a history of early-onset diabetes and features of insulin resistance, was referred at 6 weeks' gestation. Molecular testing confirmed pathogenic variants in *HNF4A* and *INSR*. Noninvasive prenatal testing predicted fetal inheritance of the *HNF4A* variant. Despite good maternal glycemic control and relatively low insulin requirements, the infant was macrosomic and developed persistent postnatal hypoglycemia requiring prolonged diazoxide therapy. This case highlights the importance of early recognition of monogenic diabetes in pregnancy, the potential utility of noninvasive prenatal testing, and the observation of persistent hyperinsulinemia beyond the neonatal period in *HNF4A*-related diabetes.

## Introduction

Maturity-onset diabetes of the young (MODY) is frequently underrecognized and misdiagnosed. It typically presents as early-onset diabetes in a nonobese individual, often with predominant postprandial hyperglycemia. Among its subtypes, hepatocyte nuclear factor 4 alpha (*HNF4A)-*MODY is characterized by progressive β-cell dysfunction. During pregnancy, *HNF4A* mutations are associated with fetal macrosomia and persistent neonatal hypoglycemia resulting from fetal hyperinsulinism. The average increase in birthweight at term has been reported as ∼800 g, independent of maternal glucose control [1].

Mutations in the insulin receptor (*INSR)* gene result in varying degrees of insulin resistance. Heterozygous mutations can present with hyperglycemia, hyperinsulinemia hypoglycemia, and clinical features such as hyperandrogenism, oligomenorrhoea, and acanthosis nigricans, even in the absence of obesity. Homozygous *INSR* mutations are associated with severe insulin resistance syndromes, characterized by intrauterine and postnatal growth restriction, dysmorphic features, and reduced life expectancy. In pregnancies affected by heterozygous *INSR* mutations, small-for-gestational-age offspring have been reported [2-4].

We present the case of a pregnant woman with coexisting *HNF4A* and *INSR* mutations, with fetal inheritance of the *HNF4A* variant. This case underscores the importance of early recognition of monogenic diabetes in pregnancy and the implications for both maternal and fetal management, including tight glycemic control, growth monitoring, and the need for ongoing metabolic surveillance of the neonate into adulthood.

## Case presentation

**Figure 1:**
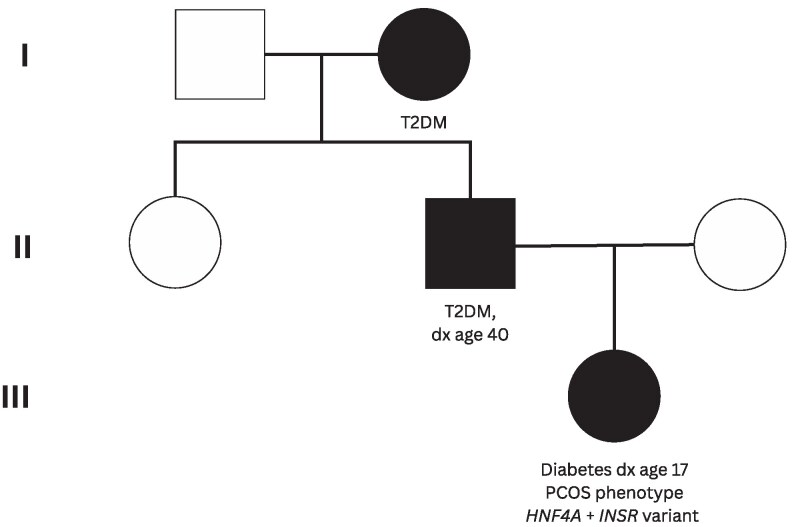
Three-generation pedigree demonstrating coexisting *HNF4A* and *INSR* variants in the proband (generation III), diagnosed with diabetes at age 17 years with a polycystic ovarian syndrome (PCOS) phenotype. Her father (generation II) had type 2 diabetes mellitus (T2DM) diagnosed at age 40 years, and her paternal grandmother (generation I) was also affected. Genetic testing was not performed in other family members. Squares indicate males, circles indicate females, and filled symbols indicate affected individuals.

A 25-year-old Caucasian gravida 1, para 0 (G1P0) woman was referred at 6 weeks' gestation to Liverpool Hospital (New South Wales, Australia). Her birth weight was 3.14 kg. She had no history of hypoglycemia during childhood. She was diagnosed with diabetes at age 17 years with a weight at diagnosis of 50 kg. Her medical history included polycystic ovarian syndrome (PCOS) with clinical features of oligomenorrhoea, hirsutism, and acne. She had a strong family history of diabetes. Her father was diagnosed with type 2 diabetes mellitus (T2DM) at age 40 years, and both paternal grandmothers were also affected. These family members to date have not been tested for *HNF4A* or *INSR* mutations (Fig. 1).

## Diagnostic assessment

Prepregnancy, her body mass index was 22.2 kg/m^2^. Laboratory tests revealed mildly elevated serum testosterone concentration of 54.7 ng/dL (SI: 1.9 nmol/L) (reference range, 5.8-52 ng/dL [SI: 0.2-1.8 nmol/L]), sex hormone binding globulin (SHBG) concentration of 2047 ng/mL (SI: 71 nmol/L) (reference range, 864-3167 ng/mL [SI: 30-110 nmol/L]), and a free androgen index of 2.7% (reference range, 0.3-4.0). She also had endometriosis and was a carrier of α and β thalassemia traits. She exhibited microcytic anemia (hemoglobin concentration of 10.4 g/dL [SI: 104 g/L] (reference range, 11.5-16.0 g/dL [SI: 115-160 g/L]) and a mean corpuscular volume of 75 fL (SI: 75 × 10^−15^ L) (reference range, 80-100 fL [SI: 80-100 × 10^−15^ L]).

At time of diagnosis, her hemoglobin A1c (HbA1c) was 6.7% (reference range, ≥6.5%), fasting plasma glucose 85 mg/dL (SI: 4.7 mmol/L) (reference range, ≥126 mg/dL [SI: ≥7.0 mmol/L]). This was likely an underestimation given her preexisting anemia and reduced red blood cell lifespan and increased cell turnover. Diabetes-related autoantibodies (glutamic acid decarboxylase, Zinc Transporter 8, islet antigen-2) were negative, and C-peptide was within normal limits (2.4 ng/mL [SI: 0.80 nmol/L]; reference range, 1.2-4.5 ng/mL [SI: 0.4-1.5 nmol/L]).

## Treatment

Prepregnancy, the patient's HbA1c was 7.3% and initially treated as T2DM with metformin 500 mg extended release twice daily. During pregnancy, her blood glucose levels (BGLs) were monitored via finger-prick capillary testing including fasting and 2-hour postprandial measurements. At 6 weeks' gestation, insulin therapy was commenced with human isophane insulin 4 units at night and insulin aspart 4 units 3 times daily. During the first trimester, fasting blood glucose levels ranged from 85 to 95 mg/dL (SI: 4.7-5.3 mmol/L); however, postprandial excursions were observed, reaching up to 205 mg/dL (SI: 11.4 mmol/L). By the end of the first trimester, her total daily insulin dose had increased to 41 units. Glycemic control was complicated by intermittent hypoglycemia (as low as 52 mg/dL [SI: 2.9 mmol/L]), necessitating frequent dose reductions to stabilize BGLs throughout the second trimester. This degree of early gestational insulin sensitivity in a lean patient was considered atypical and prompted further investigation for monogenic diabetes. A pathogenic splice-site mutation in *HNF4A* (c.320-1G > A) and a likely pathogenic *INSR* missense mutation (c.3531C > G; p.Asp1177Glu) was detected. Subsequent noninvasive prenatal testing (NIPT) predicted >95% probability of the fetus inheriting the *HNF4A* variant. The fetus was not predicted to have inherited the maternal *INSR* variant. Given fetal risk of macrosomia, the patient was recommended to undergo fortnightly fetal ultrasounds to monitor growth parameters.

## Outcome and follow-up

At 33 + 5 weeks' gestation, fasting BGL ranged from 77 to 92 mg/dL (SI: 4.3-5.1 mmol/L), with postprandial levels above target ranging from 85 to 202 mg/dL (SI: 4.7-11.2 mmol/L), despite insulin therapy (total daily dose 57 units). At 34 weeks, fetal ultrasound showed an estimated weight of 2300 g (52nd percentile) and abdominal circumference of 316.6 mm (86th percentile). The patient developed premature rupture of membranes, and labor was augmented with oxytocin because of prolonged second stage of greater than 2 hours. Delivery occurred via vacuum-assisted vaginal birth. The neonate weighed 2860 g (90th-95th percentile).

The infant was admitted to the neonatal intensive care unit for prematurity and required intravenous dextrose until day 5. Despite transitioning to enteral feeds, the mother continued to exhibit borderline hypoglycemia (BGL 54-63 mg/dL [SI: 3.0-3.5 mmol/L] with inappropriately elevated insulin of 1.8 µU/mL [SI: 11 mIU/L]; reference range, ≤1.4 µU/mL [SI: ≤9 mIU/L]), ACTH of 13 pg/mL (SI: 2.9 pmol/L) (reference range, 4.5-49 pg/mL [SI: 1.0-10.8 pmol/L]), and GH of 23.4 ng/mL (SI: 23.4 µg/L). A diagnosis of hyperinsulinemia hypoglycemia was made, and diazoxide therapy (2 mg/kg every 8 hours) was initiated from day 9 of birth. Diazoxide therapy was successfully weaned and ceased by day 25 of birth. The infant subsequently underwent a fasting challenge, which was normal. Maternal insulin was ceased postpartum because of an episode of inpatient hypoglycemia, and she was continued on metformin monotherapy because of safety considerations and breastfeeding compatibility of alternate oral hypoglycemic agents.

At 2 years postpartum, the patient's HbA1c was 6.9% with a hemoglobin of 12.2 g/dL (SI: 122 g/L) and mean corpuscular volume of 73 fL [SI: 73 × 10^−15^ L]). At 3 months of age, the infant continued to experience hypoglycemia (BGL 59-79 mg/dL [SI: 3.3-4.4 mmol/L]). Diazoxide was reintroduced at 6 mg/kg/day but was discontinued shortly afterward because of hirsutism. Blood glucose levels remained largely stable with the commencement of solid feeds at 6 months. At 28 months, the child was at the 7.9th percentile for height, 3.3rd percentile for weight. and 21st percentile for body mass index. The infant continues to have occasional low BGLs, and continuous glucose monitoring is being pursued. A repeat supervised fasting study is currently under consideration.

## Discussion

This case presents a rare antenatal scenario, not previously described (Table 1), involving dual monogenic variants: a pathogenic *HNF4A* splice-site mutation (c.320-1G > A) and a likely pathogenic *INSR* missense variant (c.3531C > G; p.Asp1177Glu), identified in a young woman with longstanding diabetes, a lean phenotype, and clinical features of insulin resistance. This case highlights the complex and potentially opposing effects these mutations can have on fetal growth and neonatal glucose regulation.

**Table 1:** Published case reports of INSR mutations in pregnancy

Case report citation	Clinical features and comorbidities	Mutation	Prepregnancy treatment	Pregnancy treatment	Fetal outcomes
Prehn et al, 2025 [5]	Diabetes (dx age 13 years) Nocturnal hypoglycemia (off insulin) PCOS/hirsutism No acanthosis nigricans or lipodystrophy Hypothyroidism Epilepsy	Heterozygous p.(Met1180Lys)	Metformin 500 mg BD	Pregnancy 1: Metformin changed to insulin at 14 weeks (mean insulin dose 36 units per day). Pregnancy 2: Metformin discontinued at 25 weeks due to IUGR. Insulin 2-6 units daily	Pregnancy 1: BW 3.95 kg, 39/40 (75th-95th percentile) No neonatal hypoglycemia Pregnancy 2: IUGR, BW 1.8 kg at 34 + 3/40 (<5th percentile) Neonatal hypoglycemia requiring IV glucose until day 2 of life
Crowley et al, 2024 [4]	Diabetes (dx age 20 years) Hirsutism Oligomenorrhoea Autoimmune thyroid disease, celiac disease and positive GAD antibodies. Overweight, no features of acanthosis nigricans.	Heterozygous missense mutation p.(Met1180Lys)	Metformin 500 mg BD	11 pregnancies treated with insulin monotherapy or combined metformin and insulin therapy. The maximum insulin dose requirement was 134 units/day late in the second pregnancy.	Pregnancy 1: Neonatal death at 21 + 2 weeks' gestation, BW 500 g Pregnancy 2: Preterm birth at 26 + 4 weeks' gestation, BW 740 g (5th percentile) Hyperinsulinemia hypoglycemia VSD, pulmonary hypoplasia Development delay Autism Pregnancy 3: BW 2870 g, 38 weeks' gestation (40th percentile) No complications Pregnancy 4: BW 3110 g, 38 weeks' gestation (66th percentile) T1DM age 2 (IA2, ZnT8 antibodies positive) Pregnancy 5: BW 3520 g (94th percentile), 38 + 3 weeks' gestation No complications Pregnancy 6/7: Twin 1 BW 2.2 kg (9th percentile) 36 + 2 Twin 2 BW 2.5 kg, fetal demise 32 weeks Pregnancy 8: BW 3.52 kg (100) 35 + 3/40, VSD, Intrauterine death
L'Aime et al, 2025 [6]	Noninsulin-requiring diabetes prepregnancy Acanthosis nigricans/possible PCOS	Deletion *INSR* c.(2029 + 1_2030-1)_(2267 + 1_2268-1)del, exons 10-11 *INSR* and *INSR* c.2936T > C, p.(Leu979Pro)	metformin 500 mg TDS	metformin 2500 mg daily, 15-26/40 TDD up to 900 IU daily	BW 3580 g, 37/40 (75th-95th percentile) Single measure of hypoglycemia after birth
Ariza Jiménez et al, 2019 [7]	Breast cancer Gestational diabetes, later diagnosed T2DM	Heterozygous alteration in exon 20 (p.Asp1177Glu, C.3531C > G)	Not reported	Not reported	Preterm, no IUGR T2DM mellitus in childhood, sporadic preprandial hypoglycemia and acanthosis nigricans.
Sethi et al, 2020 [3]	Case 1: gestational diabetes. No features of acanthosis nigricans, hirsutism, or oligomenorrhoea Case 2: hypothyroidism	Case 1: heterozygous *INSR* missense variant p.(Met1180Lys) Case 2: heterozygous *INSR* missense variant p.(Arg1119Gln) Case 3: heterozygous *INSR* p.(Arg1191Gln)	Case 1: requiring insulin in pregnancy	Not reported	Case 1: Pregnancy 1: BW 2.41 kg (−2.42 SDS), 37/40, HH requiring diazoxide until 8 months. Pregnancy 2: 2.43 kg (−2.42 SDS), 38/40. HH requiring diazoxide until 11 months. Case 2: Twin 1: BW 2.025 kg (−3.75 SDS), 36/40. HH requiring diazoxide until 10 weeks. Twin 2: 2.56 kg (−1.95 SDS). No neonatal hypoglycemia. Case 3: 2.20 kg (−1.151 SDS). 37/40. HH requiring diazoxide until 9 weeks.
Enkhtuvshin et al, 2015 [2]	No previous diagnosis of diabetes. Acanthosis nigricans.	Heterozygous deletion of nucleotides at 2995_2997 in exon 17(c.2995_2997delCTT)	None before first pregnancy. Metformin 1500 mg daily and insulin TDD 200 units	Pregnancy 1: Insulin TDD 480 units/day at 35 weeks Pregnancy 2: Metformin 2250 mg daily, insulin TDD 174 units/day	Pregnancy 1: BW 2224 g (<10th percentile), 37/40. Neonatal hypoglycemia Pregnancy 2 2532 g (<25th percentile), 38.40. Neonatal hypoglycemia
Patient (current case)	MODY Diabetes (diagnosed age 17), PCOS with oligomenorrhoea, hirsutism, and acne. Endometriosis α and β thalassemia carrier	Missense mutation c.3531C > G; p.Asp1177Glu	Metformin 500 mg XR BD	Insulin 30 units TDD by third trimester	BW 2860 g (90th-95th percentile), 34/40. Neonatal hypoglycemia into infancy.

Abbreviations: BD, twice daily; BW, birth weight; dx, diagnosis; GAD, glutamic acid decarboxylase; HH, hyperinsulinemia hypoglycemia; IA2, islet antigen 2; INSR, insulin sensing receptor; IU, international units; IUGR, intrauterine growth restriction; MODY, maturity onset of diabetes in the young; PCOS, polycystic ovarian syndrome; SDS, standard deviation score; TDD, total daily dose; TDS, ; T1DM, type 1 diabetes mellitus; T2DM, type 2 diabetes mellitus; VSD, ventricular septal defect; XR, extended release; ZnTr8, zinc transporter 8

MODY1 resulting from *HNF4A* mutations is associated with fetal hyperinsulinism, resulting in macrosomia even in the setting of well-controlled maternal glucose levels. Pearson et al (2007) reported an average 790-g increase in birthweight in mutation-positive infants, independent of maternal glycemic control. Additional risk factors of maternal hyperglycemia and insulin therapy can result in an additional increase in birth weight in addition to the direct effect of the *HNF4A* variant in the fetus. Consistent with this, our patient's fetus had an abdominal circumference in the 86th percentile at 34 weeks, with a birthweight of 2860 g at 34 + 4 weeks (90th-95th percentile), despite antenatal insulin therapy. Furthermore, this case highlights the potential role of NIPT using circulating cell-free fetal deoxyribonucleic acid in guiding antenatal care in monogenic diabetes. In this instance, NIPT confirmed likely inheritance of the maternal *HNF4A* mutation, enabling early risk stratification, serial growth monitoring, and anticipatory neonatal planning. Although promising, NIPT for monogenic conditions is not yet widely available and remains limited by cost and lack of large-scale validation [8]. Until such tools are routine, serial growth assessments every 2 weeks from 28 weeks is critical in monitoring for macrosomia and early delivery, induction of labor and/or elective cesarean section may need to be considered based on fetal size on ultrasound even with excellent maternal glucose control.

This case report also describes a novel *INSR* p.Asp1177Glu variant in the context of pregnancy [6]. Mutations associated with the *INSR* gene has been associated with type A insulin resistance in adults with prior reports describing insulin requirements ranging from very low (2-6 units before lunch) to extremely high (up to 900 units/day), reflecting variable insulin sensitivity [2-5, 7]. However, the antenatal implications of *INSR* mutations, particularly when co-inherited with *HNF4A* variants, have not previously been described. Features of hyperandrogenism, which are commonly associated with *INSR* mutations, may be modified by the presence of an *HNF4A* mutation. In *HNF4A*-related hyperinsulinemia, increased portal insulin delivery to the liver may suppress hepatic SHBG production, resulting in elevated circulating free testosterone levels and a clinical phenotype that may mimic PCOS [9]. Furthermore, our patient had unexpectedly low insulin requirements during pregnancy. *HNF4A*-MODY is primarily characterized by impaired glucose-stimulated insulin secretion rather than intrinsic insulin resistance, which may partly explain this finding [10]. Additionally, the patient's history of endometriosis and its associated chronic inflammation could contribute to mild background insulin resistance, suggesting that overall, the INSR variant may have had limited clinical impact during this pregnancy.

Metformin has been used to improve insulin sensitivity in patients; however, given the risk of small-for-gestational-age babies associated with *INSR* mutations, its use in pregnancy remains debated because of concerns regarding placental transfer and associations with reduced gestational size. Management of *INSR*-related diabetes outside pregnancy has also included glucagon-like peptide 1 receptor agonists and sodium-glucose co-transporter 2 inhibitors, with reported glycemic benefits [11]. However, these agents are not currently recommended for use in pregnancy because of limited safety data.

*HNF4A*-driven neonatal hyperinsulinism may also drive persistent hypoglycemia. Postnatally, the infant in this case exhibited transient borderline hypoglycemia, which required dextrose infusion followed by diazoxide therapy. Although *HNF4A*-positive neonates may experience transient hypoglycemia requiring short-term intravenous glucose and diazoxide therapy, most resolve within weeks to months postpartum [12, 13]. However, this duration may be highly variable, with some case reports describing the ongoing use of diazoxide therapy persisting into adolescence [14]. In addition, given the progressive β-cell dysfunction associated with *HNF4A*, carriers are also at long-term risk of developing impaired glucose tolerance and diabetes, necessitating ongoing metabolic surveillance through childhood and adolescence. Continuous glucose monitoring may be a helpful tool in these patients to identify asymptomatic hypoglycemia and hyperglycemia.

## Learning points

- MODY1 (*HNF4A*) should be considered in young, lean individuals with early-onset diabetes and a strong family history.
- Mutations in the *INSR* gene may manifest with clinical features such as hyperandrogenism, oligomenorrhoea, and acanthosis nigricans, even in the absence of obesity.
- Serial ultrasound monitoring and early delivery planning remain crucial in pregnancies affected by monogenic diabetes.
- NIPT may offer valuable early insight into fetal genotype and risk stratification when one parent is a known mutation carrier.

## Contributors

K.W. conceived and designed the case report, identified the key clinical focus, interpreted the clinical findings, contributed to the diagnostic reasoning, and drafted the manuscript. H.R. and N.R. were responsible for the diagnosis and management of the patient and critically reviewed the manuscript. All authors reviewed and approved the final draft.

## Data Availability

Original data generated and analyzed for this case report are included in this published article.

## References

[1] Pearson ER, Boj SF, Steele AM, et al Macrosomia and hyperinsulinaemic hypoglycaemia in patients with heterozygous mutations in the HNF4A gene. PLoS Med. 2007;4(4):e118. 17407387 10.1371/journal.pmed.0040118 PMC1845156

[2] Enkhtuvshin B, Nagashima S, Saito N, et al Successful pregnancy outcomes in a patient with type A insulin resistance syndrome. Diabet Med. 2015;32(6):e16‐e19. 25472847 10.1111/dme.12659 PMC5034500

[3] Sethi A, Foulds N, Ehtisham S, et al Heterozygous insulin receptor (INSR) mutation associated with neonatal hyperinsulinemic hypoglycaemia and familial diabetes Mellitus: case series. J Clin Res Pediatr Endocrinol. 2020;12(4):420‐426. 31989990 10.4274/jcrpe.galenos.2019.2019.0106 PMC7711633

[4] Crowley MT, Goulden E, Sanchez-Lechuga B, et al Case report: glycaemic management and pregnancy outcomes in a woman with an insulin receptor mutation, p.Met1180Lys. Clin Diabetes Endocrinol. 2024;10(1):5. 38461278 10.1186/s40842-024-00166-9 PMC10924971

[5] Prehn EL, Crowley M, Fennell D, Kinsley BT, Colclough K, Byrne MM. Pre-gestational diabetes in a young woman with a pathogenic INSR missense mutation, p.(Met1180Lys). Endocrinol Diabetes Metab Case Rep. 2025;2025(1):e240087. 39922186 10.1530/EDM-24-0087 PMC11825164

[6] L'Amie AK, Hall RM, Bate J, Cox S, Murphy R. Two cases of severe insulin resistance in pregnancy: a new diagnosis of INSR variant and a patient with SHORT syndrome. JCEM Case Rep. 2025;3(11):luaf243. 41140512 10.1210/jcemcr/luaf243 PMC12550683

[7] Ariza Jiménez AB, López Siguero JP, Martínez Aedo Ollero MJ, Del Pino de la Fuente A, Leiva Gea I. Mutación del gen INSR: insulinorresistencia poco prevalente en edad pediátrica, a propósito de un caso. Endocrinol Diabetes Nutr. 2019;66(9):588‐589. 10.1016/j.endinu.2019.04.005 31229400

[8] Abedalthagafi M, Bawazeer S, Fawaz RI, Heritage AM, Alajaji NM, Faqeih E. Non-invasive prenatal testing: a revolutionary journey in prenatal testing. Front Med (Lausanne). 2023;10:1265090. 38020177 10.3389/fmed.2023.1265090 PMC10666054

[9] Plymate SR, Matej LA, Jones RE, Friedl KE. Inhibition of sex hormone-binding globulin production in the human hepatoma (Hep G2) cell line by insulin and prolactin. J Clin Endocrinol Metab. 1988;67(3):460‐464. 2842359 10.1210/jcem-67-3-460

[10] Gulisano C, Aloi C, Salina A, et al Case report: beyond type 1 diabetes: a case of delayed MODY1 diagnosis and successful transition to sulfonylurea therapy. Front Med (Lausanne). 2025;12:1590935. 40486195 10.3389/fmed.2025.1590935 PMC12141343

[11] Martínez-Montoro JI, Pinzón-Martín JL, Damas-Fuentes M, Fernández-Valero A, Tinahones FJ. Combination therapy with semaglutide and dapagliflozin as an effective approach for the management of type A insulin resistance syndrome: a case report. Front Endocrinol (Lausanne). 2022;13:838887. 35498407 10.3389/fendo.2022.838887 PMC9046779

[12] Kapoor RR, Locke J, Colclough K, et al Persistent hyperinsulinemic hypoglycemia and maturity-onset diabetes of the young due to heterozygous HNF4A mutations. Diabetes. 2008;57(6):1659‐1663. 18268044 10.2337/db07-1657

[13] Fajans SS, Bell GI. Macrosomia and neonatal hypoglycaemia in RW pedigree subjects with a mutation (Q268X) in the gene encoding hepatocyte nuclear factor 4alpha (HNF4A). Diabetologia. 2007;50(12):2600‐2601. 17891372 10.1007/s00125-007-0833-7

[14] McGlacken-Byrne SM, Hawkes CP, Flanagan SE, Ellard S, McDonnell CM, Murphy NP. The evolving course of HNF4A hyperinsulinaemic hypoglycaemia–a case series. Diabet Med. 2014;31(1):e1‐e5. 23796040 10.1111/dme.12259

